# Introduction—Centro de Astrobiología: 20 Years Building Astrobiology

**DOI:** 10.1089/ast.2020.0804

**Published:** 2020-09-15

**Authors:** Víctor Parro, J. Miguel Mas-Hesse, Javier Gomez-Elvira, Álvaro Giménez, Juan Pérez-Mercader

**Affiliations:** ^1^ Centro de Astrobiología (CAB, CSIC-INTA), Torrejón de Ardoz, Madrid, Spain.; ^2^ Department of Payloads and Space Sciences (INTA), Torrejón de Ardoz, Madrid, Spain.; ^3^ Department of Earth and Planetary Sciences and Origins of Life Initiative, Harvard University, Cambridge, Massachusetts, USA.

## The Origins

The Centro de AstroBiología (CAB) was founded in November 1999 as a joint institute between the Spanish National Research Council (CSIC) and the National Institute for Aerospace Technologies (INTA). Located in Madrid (Spain), CAB became the first astrobiology organization outside the United States to be associated with the NASA Astrobiology Institute (NAI)—formally becoming an *associate* member in the year 2000. Astrobiology considers life as a natural consequence of the evolution of the Universe, and CAB aims to study the origin, evolution, distribution, and future of life in the Universe, from an integrative transdisciplinary approach.

CAB's foundation was the result of a profound interest in applying the scientific method to life—in the sense of looking for or making another form of “life” to compare it with life on Earth—to therefore infer the principles that were behind the class of phenomena we encounter in biology. Discussions on these topics started in Santa Fe and Los Alamos, New Mexico. They originally (1993) focused on finding a common phenomenology to self-organized phenomena from the Big Bang to biology and on establishing some “bridge” between the Big Bang and biology. They involved Murray Gell-Mann, Geoffrey West, and Juan Pérez-Mercader, who was to be the founding director of CAB. They focused on looking at the ubiquitous power laws present at every scale in the Universe. These power laws are indicators of self-organization in collective phenomena, and they indicate collective states where the components somehow contribute “equally” to the generation of a cooperative state, just as in phase transitions in physics. The big question could be framed as follows: Are the various collective phenomena that seem to have formed in a “successive” way through the history of the Universe the result of “phase transitions,” and was their coarse-graining provided by the “arrow of time”? There are many harbingers for this. But establishing the correlation is not trivial for many reasons, including the fact that we only know one example of life, and we do not know how it was generated. It would require a team of scientists with a truly scientific and professional knowledge and understanding of the various components of the puzzle.

That team began to emerge in Madrid, with scientists working in galactic astrophysics, planetary science, evolution of life and of viruses, origins-of-life chemistry, metabolic processes in biochemistry, evolution of planets, bioinformatics, extremophiles, and physics, together with engineers to design and produce instrumentation. This group, from a variety of universities and government research centers, such as INTA (Instituto Nacional de Técnica Aeroespacial) and CSIC (Consejo Superior de Investigaciones Científicas), started informal discussions; and a passion for the subject of astrobiology began to burn. Then exoplanets were discovered in 1995, a report of fossil-like objects in the ancient martian meteorite ALH84001 came out in 1996, and NASA's Pathfinder mission with the little rover Sojourner landed on Mars in 1997. It looked like a dream come true. NASA was also very interested in the resurgence of the subject; and conversations with the late Jerry Soffen, Carl Pilcher, Michael Meyer, Scott Hubbard, and many others started. They all were full of inspiration, enthusiasm, and collegial exchanges. They also were tremendously helpful and generous. The NAI was formed, and by 2000 CAB was officially an Associate Member. We felt a real part of it and loved the institution and what it meant and represented. Collaboration with other European groups began to form as well, and soon we were cooperating with the Beagle-2 probe to Mars.

From day zero, the Centro de Astrobiología had the support of the Government of Spain and the European Union, with whose help the Centro began to hire new postdocs in the areas of science where astrobiology had a more intense focus. The timing was perfect, and many good things followed. New ideas for instruments for the detection of life in arbitrary environments began to be discussed, and proposals were written. Prototypes were built and tested in the field. The Río Tinto attracted astrobiologists from around the world and generated a great influx of visitors and collaborators. More diverse experiments and projects followed, and younger members of CAB began to see that they could work in these topics and be immersed in a truly international collaboration where their ideas and those of their mentors were tested. With time, they began to compete for resources and build instrumentation and do observational field campaigns in many places on Earth—and eventually outside Earth, for example with NASA on board the Curiosity and Perseverance rovers.

The main idea was to have theoreticians, working with computer simulation experts, observational scientists, and experimentalists. These represent the four pillars of the contemporary scientific method, all in collaboration with engineers. Inspired by NASA's outreach and education program, we also began to popularize aspects of our science programs with that we felt could inspire the general public. These projects had “life” at their center, as if it was the palm of a hand. And persons in different disciplines, represented by the fingers, would look at the problem in the “palm” with the tools and methods of the particular discipline. Then a logical discussion of what was seen in the “palm” would take place between the “fingers.” The need for consensus and logical explanations of what the various sciences encountered constituted our “transdisciplinary” experience. It was fantastic and still is.

## Vision and Mission

Astrobiology challenges science and the scientific community to develop and apply new methods and approaches. We regard astrobiology as a scientific discipline in its own right that requires a transdisciplinary approach involving theory, simulation, observation, instrument development, and experimentation. How to apply fundamental scientific principles to answer the question (or questions) of astrobiology is the most important challenge for CAB. The unique transdisciplinary setting at CAB creates the appropriate atmosphere to allow engineers to interact with experimental, theoretical, and observational scientists from various fields: astronomy and astrophysics, geology, biogeochemistry, microbiology, genetics, remote sensing, microbial ecology, computer science, physics, robotics, and communications engineering. The research at CAB is focused on specific questions related to the systematization of the chain of events that took place between the Big Bang and the origin, evolution, and distribution of life, including the self-organization of interstellar gas into complex molecules. We are interested in understanding key phenomena that might affect life's origin and evolution from galactic environments, stars, and planet formation and evolution, and the complexity of the chemistry in interstellar space and mature prebiotic chemistry in planetary environments. Among CAB's most relevant lines of research are planetary scenarios that can create and evolve life as we know it; the versatility and plasticity of hypothetical biochemistry and metabolisms in different planetary environments; and the heart of astrobiology via the investigation of the possibility of life on other worlds.

## Organization

To address these fundamental questions, research at CAB is focused on the following areas:

- Formation and evolution of galaxies, the interstellar medium, stars, and planets: Extragalactic and stellar astrophysics, comparative planetology, and molecular astrophysics.
- Molecular evolution and adaptation: Prebiotic chemistry; molecular adaptation of replicative systems; metabolic and environmental-dependent molecular adaptation; molecular biomarkers.
- Evolution and characterization of potential habitable environments in the Solar System: Geomicrobiology of extreme environments, planetary geology and atmospheres, and environmental and biological signatures.
- Development of advanced instrumentation: Astronomical instrumentation and instrumentation for *in situ* planetary exploration; ground simulation chambers.

The main CAB building and facilities are located within the INTA campus in Torrejón de Ardoz, and a second section is in ESA's European Space Astronomy Centre (ESAC), both close to Madrid. INTA, the reference Spanish institution in aerospace technology, with nearly 80 years of existence, offers CAB a unique environment where synergies emerge easily. Similarly, the close contact with ESA's Astronomy Centre makes the involvement of our researchers in ESA's missions a natural and fluent process.

Currently, CAB has 155 workers that include 55 civil servants, 75 contract employees, and 25 PhD students. We are functionally organized into four departments: Astrophysics, Molecular Evolution, Planetology and Habitability, and Advanced Instrumentation. Several laboratories are equipped with facilities and instrumentation for multiple techniques, such as biochemistry, molecular ecology, DNA sequencing, microbiology, geology, geomineralogy, geochemistry, organic chemistry, stable isotope analysis, nano-dispensing and microarray capabilities, facilities for meteoritic impact simulation, high-vacuum simulation chambers, high-pressure chambers, a Mars wind tunnel and an environmental chamber, and a cryostat for IR detector development for astronomical applications. The highly multidisciplinary team (astrophysicists, planetologists, geologists, geochemists, chemists, geomicrobiologists, molecular microbiologists, engineers, and system engineers), together with the diversity of facilities and techniques available, make CAB a unique research center where multidisciplinarity turns into true transdisciplinarity to tackle astrobiological questions.

## Main Scientific Achievements

In our first 20 years, CAB scientists have published their research and achievements in more than 2800 articles (source: Scopus) in international journals, the majority of which lie within the first quartile (Q1) with regard to impact factor. Much of this scientific production is a consequence of a strong international collaboration with colleagues from European and American institutions. The main contributions are related to (i) understanding the extremes of life and its feasibility in other worlds through the study of terrestrial analogs of other planetary environments; (ii) the development of instrumentation for *in situ* planetary exploration, either to characterize the martian environment with environmental sensors, or *in situ* detection of biomarkers; (iii) contribution to the science and building of astronomical instrumentation that allows, among other objectives, the detection and characterization of exoplanets; and (iv) building ground simulation facilities to understand planetary atmospheres on Earth or the complexity of chemistry in interstellar space.

### Terrestrial analogs and the Solar System

Understanding the extremes of life was one of the key drivers that emerged during the founding of CAB. Researchers from CAB have traveled to explore remote extreme environments around the world, studying their microbial and metabolic diversity, and how life has adapted to extreme parameters such as low pH, freezing or boiling temperatures, extremely low desiccation, high salt concentration, or minimal nutrient availability. Notable is research by CAB scientists focused on the adaptation of life to extremely low pH and high metal concentrations, especially iron. The Río Tinto system, a 90 km permanent stream with a constant pH of 2.3 along its entire length, is the paradigm for studying life based on iron and sulfur chemistry, as well as a good terrestrial analog for early wet and acidic areas on Mars. We have explored from the Río Tinto waters and shallow sediments to the deep subsurface (600 m depth) of the rocky Iberian Pyrite Belt (IPB) massive sulfide ore, thanks to the European Research Council (ERC) Advanced Grant IPBSL. Our studies revealed extremely acidic waters with an unexpected microbiological diversity that adapts to poly-extreme conditions (low pH, high iron and sulfur concentrations, high oxidative stress, oligotrophy) through a variety molecular mechanisms. We have learnt that even cyanobacteria are alive and viable in the deep and dark rocky subsurface and that full iron, sulfur, carbon, and nitrogen cycles are operating in the depths of the IPB.

Two recent ERC-funded projects allow us to tackle key investigations about Mars. A *Starting Grant* entitled “A cold and wet early Mars” helped solve the decades-old problem of the early martian climate, proposing and testing a new hypothesis to understand the early environmental traits on Mars. With a *Consolidator Grant* we aim to provide a precise understanding of the inventory of water during the first billion years of martian history and its early evolution on both global and local scales. CAB is also interested in the outer Solar System and participates in ESA's JUICE mission as well as simulating ocean world conditions in ground simulation facilities.

### Development of instrumentation for *in situ* planetary exploration

The Río Tinto area remains an ideal site for instrument development and maturation for planetary exploration. Early in CAB's history, the MARTE project (Mars Analogue Research and Technology Experiment), a CAB-NASA Ames Research Center collaboration, was an important milestone that initiated a fruitful collaboration between both centers with regard to the development of a future robotic drilling platform with payload instrumentation that would enable life-detection missions to explore the martian subsurface. One essential element of this payload was SOLID (Signs of Life Detector), a sample processing and analytical instrument capable of detecting molecular biomarkers by means of an antibody microarray-based biosensor, the so-called LDChip (Life Detector Chip). SOLID-LDChip has been tested and validated in multiple field campaigns (Río Tinto, Antarctica, Atacama Desert, the High Arctic, deep South African mines, and the Antarctic Ice Sheet) and has detected microbial markers at each site with the use of its more than 200 interrogating probes.

CAB also committed early in its history to develop, deliver, and operate the REMS (Rover Environmental Monitoring Station) environmental station for NASA's Mars Science Laboratory (MSL) mission. REMS has successfully relayed information from the Curiosity rover back to Earth since 2012, providing the environmental information required to understand the martian atmosphere and its dynamic processes at Gale Crater. Since November 2018, the TWINS (Temperature and Winds for INSight) instrument, via the heritage of REMS, has provided valuable information about the martian atmosphere at the Insight landing site, as well as support for other instruments and experiments of the mission. Finally, a more sophisticated set of environmental sensors led by CAB makes up the MEDA (Mars Environmental Dynamics Analyzer) instrument for NASA's Mars 2020 mission. Installed on the Perseverance rover, MEDA will make environmental measurements including air and ground temperature, pressure, UV radiation, and atmospheric opacity. Besides providing critical information to other instruments, MEDA will contribute to one of the main objectives of the mission: to prepare for human exploration by providing daily reports about the evolution of the martian atmosphere.

### Astronomical research

CAB scientists are also playing a significant role in the development of astronomical instrumentation, both for ground-based and, especially, for space observatories. INTEGRAL/OMC, Cheops, and PLATO are high-accuracy optical photometers on ESA missions, the last two devoted to the identification and characterization of exoplanetary systems. Indeed, PLATO (with a CAB scientist as co-Principal Investigator) will be the first mission optimized to detect and characterize Earth-like planets in the habitable zone of solar-like stars from 2026 onwards. CAB has also had significant involvement in the CARMENES instrument on the Calar Alto observatory (Almería, Spain), the first radial velocity spectrometer working in the infrared and optimized to identify exoplanets around dwarf stars. We are also contributing to the development of the HARMONI optical and near-IR integral field spectrograph for the Extremely Large Telescope, which will be capable of detecting a range of objects from extrasolar planets to high redshift galaxies with unprecedented sensitivity and angular resolution. In the next decade, with the development of SPICA—a powerful IR telescope developed in collaboration between ESA and JAXA—another CAB researcher will serve as co-Principal Investigator of its SAFARI Far-IR Spectrometer. Finally, CAB hosts the Spanish Virtual Observatory and manages the data archives of the 10 m GTC (Gran Telescope of the Canaries) and the Calar Alto telescopes, which are available for use by the entire international scientific community.

### Ground simulation facilities

Space research performed at CAB in the laboratory through experimentation and simulation includes the chemistry of the interstellar medium and planetary atmospheres, as well as the search for potentially habitable environments. We applied vacuum and high-pressure technology to design simulation facilities appropriate for studying the physical, chemical, and biological changes induced in a particular sample by *in situ* irradiation or physical parameters in a controlled environment. Furthermore, the implementation of several spectroscopies in our simulation chambers, such as IR, Raman, and UV to study solids, and mass spectrometry to monitor the gas phases, provides specific tools for *in situ* physicochemical characterization of analogs of astrobiological interest. Our innovative designs and engineering capabilities have led to the construction of (i) ISAC (InterStellar Astrochemistry Chamber), an ultra-high-vacuum setup with a base pressure of 2.5–4.0 × 10^−11^ mbar, designed to grow thin ice films that are compositional analogs for the interstellar and precometary ice mantles that coat dust grains. Detailed study of the physical and chemical properties of ice with ISAC provided a basis for observational interpretations of comets during the Rosetta mission and protoplanetary disks using ALMA (Atacama Large Millimeter/submillimeter Array). (ii) PASC (Planetary Atmospheres and Surfaces Chamber) is an ultra-high-vacuum chamber capable of reproducing atmospheric compositions and surface temperatures representative of most planetary objects in our solar system. Experiments have been designed to simulate processes under martian conditions, study the UV photocatalytic effect on minerals related to prebiotic chemistry processes, and detect and sequester with CAB-designed nanoparticle sensors specific atmospheric components. (iii) HPPECs (High-Pressure Planetary Environment Chambers) can simulate pressure up to 10,000 bar, which enables simulation of the physicochemical processes that take place in icy moons that may host an ocean under the ice. Novel data of ice mixture properties are obtained using this facility to interpret surface features and radar measurements from missions such as JUICE and Europa Clipper to Ganymede and Europa. (iv) MARTE (Mars simulation chamber) allows simulation of the martian atmosphere to test environmental sensors and other instruments to increase their Technology Readiness Level (TRL) or to carry out experiments focused on understanding the effect of martian conditions on biological systems, or on physicochemical processes such the phase formation-deliquescence phenomena.

This issue of *Astrobiology* contains four articles that illustrate the breadth of research at CAB: (i) Jiménez-Serra *et al.* (in this issue) explore the presence of precursors and key prebiotic species in the RNA-world hypothesis in space through deep and high-sensitivity observations of the interstellar medium and of a proto-Sun. (ii) García-Descalzo *et al.* (in this issue) report experiments that illustrate how halophilic and psychrophilic microbes may alter the melting and freezing temperature of salty solutions and discuss the potential significance for the field of astrobiology. (iii) Sánchez-García *et al.* (in this issue) illustrate the heterogeneity of vertical biomarker profiles when sampled randomly during a Mars simulation drilling campaign in the terrestrial analog of Río Tinto sediments. This underscores the need for a suite of suitable analytical techniques for biomarker detection in planetary exploration as those used in this work for ground-truthing and validating samples collected with a prototype drill for Mars exploration. (iv) Finally, Fairén *et al.* (in this issue) describe a new instrument concept where microscopy, vibrational fingerprints of Raman spectroscopy, and the plasticity of macromolecular-affinity-based sensors are used together to detect chemical complexity that could be of biological origin, either for Mars or icy moon exploration.

## The Future

The hard work and breadth of the successful projects of the past 20 years at CAB have made it possible for us to be actively engaged in the main international space projects and missions. Our diverse portfolio of accomplishments has been acknowledged by the Spanish Ministry of Science via a “María de Maeztu” Scientific Excellence award that funded the project “Assessing the feasibility of life as a universal phenomenon through planetary exploration.” This project is making it possible for us to reinforce and strengthen interactions between current research directions at CAB and, at the same time, promote the growth of new areas of research that will develop into new transdisciplinary and impactful projects.

The coming decade is full of new challenges and opportunities for the Centro de Astrobiología. CAB scientists will have an unprecedented opportunity to follow the martian atmosphere simultaneously from three separate environmental stations that were built and are or will be operated under their leadership (REMS, TWINS, and MEDA). CAB scientists and engineers will also contribute to the operation of the RLS (Raman Laser Spectrometer) that will fly on ESA's ExoMars mission which will deliver the Rosalind Franklin rover on Mars (to be launched in 2022).

The advent of new and more sophisticated space instrumentation, either in space telescopes or ground-based ones, will open new avenues for space research that CAB scientists are pursuing. Particularly relevant will be ESA's PLATO mission that will search and characterize terrestrial-like planets and their atmospheres. Understanding the potentially habitable environments of the thousands of planets to be discovered, along with deciphering their remotely detected biomarkers, requires more analog research here on Earth in terms of improving our ability to understand how life can live and interact with its environments on the surface and in the deep subsurface. CAB has the means, the facilities, the scientists, and a nice building to tackle and contribute to the main challenges of astrobiology in the near future.

